# The influence of platelet-rich fibrin combined with hydroxychloroquine on the inflammatory response; bone metabolism; and cell differentiation ability in temporomandibular joint osteoarthritis

**DOI:** 10.5937/jomb0-60052

**Published:** 2026-03-17

**Authors:** Yuhan Zhao, Hongwei Dai

**Affiliations:** 1 Chongqing Medical University, College of Stomatology, China; 2 Chongqing Key Laboratory for Oral Diseases and Biomedical Sciences, China + Chongqing Municipal Key Laboratory of Oral Biomedical Engineering of Higher Education, China

**Keywords:** temporomandibular joint osteoarthritis, platelet-rich fibrin, inflammatory factors, cell differentiation, coagulation function, bone metabolism, Osteoartritis temporomandibularnog zgloba, fibrin bogat trombocitima, inflamatorni faktori, celijska diferencijacija, koagulaciona funkcija, metabolizam kostiju

## Abstract

**Background:**

This study aims to explore the regulatory effects of platelet-rich fibrin (PRF) on serum inflammatory response, bone metabolic balance and cell differentiation ability in patients with temporomandibular joint osteoarthritis (TMJ-OA) at the molecular level.

**Methods:**

Sixty patients were randomly divided into Group A and Group B (30 cases in each group). Group A was treated with meloxicam dispersible combined with hydrox-ychloroquine sulfate (HCQ); Group B was supplemented with autologous PRF intra-articular injection on the basis of Group A. Fasting venous blood was collected before treat-ment and 3 months after treatment. Inflammatory and bone metabolism markers such as IL-ip, IL-6, TNF-a, OPG, OPN, ALP and Runx2 were detected by e L iSA, and coagulation indicators such as A PTT and PT were deter-mined by an automatic coagulation analyzer.

**Results:**

After treatment, the inflammatory factors (IL-ip, IL-6, TNF-a) in both groups decreased significantly, but the decrease in group B was more significant (P&lt; 0.05). Bone metabolism indicators showed that the improvement in group B was significantly better than that in group A (P&lt; 0.05). Among the cell differentiation-related factors, the expression levels of Runx2, TGF-pi and Sox9 in group B were significantly increased compared with those before treatment (P&lt; 0.05), and were higher than those in group A (P&lt; 0.05).

**Conclusions:**

The use of PRF can optimize the therapeutic effect of TMJ-OA, improve inflammatory responses, promote cell differentiation and improve coagulation function parameters.

## Abstract (sr)

**Uvod:**

Cilj ove studije je da istraži regulatorne efekte fibrinabogatog trombocitima (PRF) na serumski inflamatorniodgovor, metabolički balans kostiju i sposobnost celijskediferencijacije kod pacijenata sa osteoartritisom temporomandibularnog zgloba (TMJ-OA) na molekularnom nivou.

**Metode:**

Sezdeset pacijenata je nasumično podeljeno ugrupu A i grupu B (po 30 slučajeva u svakoj grupi). GrupaA je lečena disperzibilnim meloksikamom u kombinaciji sahidroksihlorokin sulfatom (HCQ); Grupa B je dopunjenaautolognom PRF intraartikularnom injekcijom na osnovugrupe A. Venska krv na gladno je prikupljena pre tretmanai 3 meseca nakon tretmana. Inflamatorni i metaboličkimarkeri kostiju kao sto su IL-ip, IL-6, TNF-a, OPG, OPN,ALP i Runx2 su detektovani ELISA testom, a indikatorikoagulacije kao sto su A P TT i PT su određeni automatskimanalizatorom koagulacije.

**Rezultati:**

Nakon tretmana, inflamatorni faktori (IL-ip , IL-6,TNF-a) su se značajno smanjili u obe grupe, ali je smanjenje u grupi B bilo značajnije (P&lt; 0,05). Indikatori metabolizma kostiju pokazali su da je poboljsanje u grupi B biloznačajno bolje nego u grupi A (P&lt; 0,05). Među faktorimapovezanim sa celijskom diferencijacijom, nivoi ekspresijeRunx2, TGF-pi i Sox9 u grupi B su bili značajno povecaniu poređenju sa onima pre tretmana (P&lt; 0,05) i bili su visinego u grupi A (P&lt; 0,05). Sto se tiče koagulacione funkcije, A PTT i PT u grupi B su bili produženi (P&lt; 0,05), a nivoiFIB i D-D su se smanjili (P&lt; 0,05).

**Zaključak:**

Upotreba PRF može optimizovati terapeutskiefekat TMJ-OA, poboljšati inflamatorne odgovore, promovisati diferencijaciju celija i poboljšati parametre koagulacione funkcije.

## Introduction

Temporomandibular joint osteoarthritis (TMJ-OA), an important branch of osteoarthritis characterized primarily by subchondral bone remodeling, progressive cartilage degeneration, synovitis, and chronic pain, is often accompanied by destruction of the mandibular condyle and articular fossa, including severe pain and joint dysfunction caused by erosive absorption, sclerosis, osteophyte formation, and wear. TMJ-OA has a complex pathogenesis, which is often considered to be caused by chondrocyte apoptosis and local inflammation. The treatment for TMJ-OA is mainly to prevent further destruction of condylar cartilage and subchondral bone, relieve pain, reduce adverse load, and promote joint functional recovery [1][2]. Hydroxychloroquine sulfate (HCQ) is clinically applied to the treatment of various arthritis, which can relieve joint pain by inhibiting prostaglandin synthesis and reducing inflammatory exudation [3]. With the further clinical exploration of TMJ-OA, biological supplements have become the latest concept for the treatment of this disease. Platelet-rich fibrin (PRF), derived from platelet concentrate, is effective in accelerating tissue regeneration, promoting osteogenesis, combating infections, and accelerating wound healing, which is therefore widely used in the department of stomatology [4][5].

Currently, while the application of PRF in treating oral diseases is fairly common [6][7], most of these studies tend to concentrate on observing its therapeutic effects, with relatively little exploration into the underlying mechanisms through which PRF exerts such actions. Moreover, despite the presence of some preliminary research investigating PRF's impact on the inflammatory response and immune regulation of mesenchymal stem cells (MSCs) [8][9], its specific mechanism in treating TMJ-OA remains poorly understood.

From the perspective of serum inflammation and bone metabolism, this study examines PRF's influence on TMJ-OA by detecting changes in serum inflammatory factors and bone metabolism parameters pre- and post-treatment, so as to provide a more reliable and comprehensive reference and guidance for the clinical application of PRF.

## Materials and methods

### Included subjects

Period: January 2021 to January 2023; Subjects: 60 patients with TMJ-OA. Sample size estimation was performed using G*Power 3.1 software. Based on preliminary data from 10 pilot patients, the expected effect size (Cohen's d) for the primary outcome measure, the change in serum IL-6 levels from baseline to 3 months post-treatment, was estimated to be 0.9. With a significance level (α) of 0.05 (two-tailed) and a desired power (1-β) of 0.80, the required sample size per group was calculated to be 21. To account for a potential 20% dropout rate, the final target sample size was set to 26 participants per group. Considering clinical feasibility and to enhance statistical robustness, we aimed to recruit 30 patients per group, totaling 60 participants. Participants were randomly assigned using a computer-generated random number sequence (block randomization, block size=4) to either group A or group B, with 30 cases in each group. The balance of two groups of baseline information is ideal (P>0.05) (Table 1). The study was approved by the Ethics Committee of Chongqing Medical University.

**Table 1:** Comparison of baseline information.

Baseline information		Group A (n=30)	Group B (n=30)	*t/x^2^/Z*	P
Gender	Male	12	15	x^2^=0.606	0.436
	Female	18	15		
Age (years)		35.23±3.05	35.33±3.10	t=0.126	0.900
Duration of pain (weeks)		5.05±0.52	5.11±0.50	t=0.456	0.650
Affected side	Unilateral	15	12	x^2^=0.606	0.436
	Bilateral	15	18		
Dworkin stage	Stage I	1	2	Z=0.186	0.852
	Stage II	4	3		
	Stage III	22	21		
	Stage IV	3	4		
Course of disease (weeks)		15.12±1.52	15.15±1.55	t=0.076	0.940
Educational level	High school or below	10	8	x^2^=0.318	0.573
	Junior college or above	20	22		
Body mass index (kg/m^2^)		24.85±0.23	24.72±0.25	t=2.096	0.041

### Inclusion and exclusion criteria

Inclusion criteria: 1) The diagnosis of TMJ-OA was made by referring to the diagnostic criteria in the reference [10] and any of the following signs were observed through cone-beam computed tomography: Concave absorption of bone pits, cystic changes, sclerotic changes of bone, burr-like or osteophyte formation; 2) First time visiting for TMJ-OA; 3) No disease-related treatment before enrollment; 4) All enrolled subjects knew the purpose of the study and signed consent forms.

Exclusion criteria: 1) Patients with easily allergic constitution; 2) Patients with eye diseases such as retinopathy and maculopathy; 3) Patients with decreased renal function; 4) Female during lactation, gestation or menstrual period; 5) Patients with diseases of the coagulation system, nervous system and immune system; 6) Patients with malignant diseases; 7) Patients with mental disorders or low compliance cannot cooperate with the study to smoothly carry out; 8) Patients with hematological system or blood-related diseases; 9) History of immunosuppressive or anticoagulant therapy within 1 month before enrollment; 10) Patients with temporomandibular joint trauma history; 11) Laboratory tests were positive for rheumatic disease factors.

### Methods

Autologous PRF Preparation: Venous blood (approximately 10 mL per patient) was drawn from the antecubital vein into sterile 10 mL glass-coated plastic tubes (without anticoagulant). The tubes were immediately centrifuged at 2700-3000 rpm (approximately 400 g) for 12 min using a standardized centrifuge. Following centrifugation, the blood separated into three layers: an upper acellular plasma layer (platelet-poor plasma, PPP), a middle PRF, and a bottom red blood cell layer. The PRF clot was carefully separated from the PPP and RBC layers using sterile forceps and scissors, and transferred to a sterile metal cup. The fibrin clot was then compressed using sterile gauze to expel excess fluid, forming a solid PRF membrane. Approximately 1 mL of the compressed PRF gel/membrane was prepared for injection per joint.

Group A: Patients were given meloxicam dispersible tablets (Hainan Quanxing Pharmaceutical Co. Ltd., H20020245) 7.5 mg/time orally, 1 time /d + HCQ (FUAN Pharmaceutical Group Chongqing Bosen Pharmaceutical Co. Ltd., H20173356) 200 mg/time orally, 2 times /d, and the total treatment was 2 weeks.

Group B: On the basis of group A, autologous PRF was given. Disinfection of the pre auricular area of the affected side, needle entry point: The continuous surface projection on the plane of easy-to-orbit ear was the body surface. At the depression between 1cm in front of tragus and ankle-like process, a 10 mL syringe was used to inject 0.5 mL local anesthesia with 2% lidocaine subcutaneously. The patient was asked to open his mouth half, and the direction of needle insertion was forward, upward and inward. The needle was inserted at a depth of 3 cm, until it reached the bone surface of the articular fossa. No blood was pumped back, and 1 mL autologous PRF was injected. All procedures were performed under CBCT guidance. The patients in the two groups were injected once every two weeks, with three times as a course of treatment, for a total of three months.

### Laboratory tests

5 milliliters of fasting venous blood were collected and centrifuged (3000 r/min, r: 10 cm) for 10 min in a centrifuge manufactured by Beckman Coulter, the US; Enzyme-linked immunosorbent assay (Kit: Wuhan Saipei Biotechnology Co., Ltd.) was used to detect serum interleukin (IL)-1β, IL-6, and tumor necrosis factor-α (TNF-α), osteopontin (OPN), Osteoprotegerin (OPG), ALP transforming growth factor-β1 (TGF -β1), Runx2, and Sox9. 100 μL of IL-1β standard or sample (diluted at a 1: 2 ratio) was added to each well of a 96-well plate, followed by overnight coating at 4°C. On the following day, the coating solution was discarded, and 200 μL of blocking solution (1% BSA/PBST) was added to each well. After this step, 100 μL of biotinylated anti-antibody (provided in the kit) was dispensed into each well and incubated at 37°C for 1 hour, followed by the addition of streptavidin-HRP at 100 μL per well. The reaction was terminated by adding 50 μL of 1 mol/L H_2_SO_4_. Immediately afterward, the optical density (OD) value at 450 nm was measured using a microplate reader.

An automatic Coagulation Function Analyzer (Sysmex CS-5100) was used to detect APTT, PT, TT, FIB, and D-D concentrations. The testing procedure involved mixing 100 μL of plasma with 50 μL of the supporting reagent, preheating the mixture at 37°C for 3 min, then adding 50 μL of 0.025 mol/L CaCl_2_. The test was initiated once the computer system was activated, and the results were recorded accordingly.

### Quality control

For ELISA: A standard curve (0, 50, 100, 200, 400 pg/mL) was used for weekly calibration to maintain consistent OD value linearity. For the Coagulation Analyzer: The instrument was calibrated monthly with the manufacturer-matched calibrator (Sysmex CS-5100 Calibrator Set), with subsequent internal quality control verification.

Daily testing included low and high-value quality control samples, tracked using Levey-Jennings charts for mean, standard deviation (SD), and coefficient of variation (CV). Performance criteria specified intraassay CV limits of 5% (ELISA) and 3% (coagulation), while inter-assay CV should not exceed 10% (ELISA) or 5% (coagulation).

### Statistical methods

SPSS 24.0 software was selected to process the data. Measurement data were described as (χ) (the Shapiro-wilk test was used to confirm that the data conformed to a normal distribution), and the independent sample *t*-test and paired *t*-test were used. The count data were expressed as percentages using the χ^2^ test, the rank data were tested using the Mann-Whitney U test (for independent samples) or the Wilcoxon signed-rank test (for paired samples), as appropriate, and the test level was α=0.05.

## Results

### Comparison of inflammatory reactions

**Figure 1:**
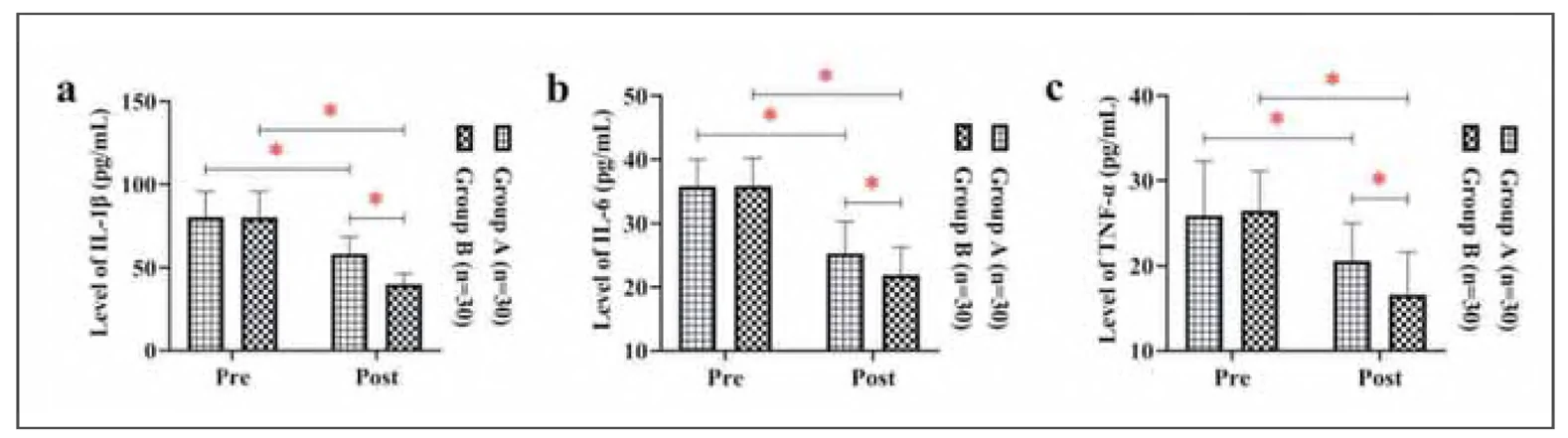
Comparison of inflammatory factors between the two groups. *P < 0.05. (a) Changes of IL-1β before and after treatment; (b) Changes of IL-6 before and after treatment; (c) Changes of TNF-α before and after treatment.

First of all, the detection of inflammatory reactions revealed reductions in IL-1β, IL-6, and TNF-α in both groups post-treatment (P<0.05), with lower levels in group B versus group A (P<0.05), suggesting a milder inflammatory response in group B (Figure 1).

### Bone metabolism comparison

**Figure 2:**
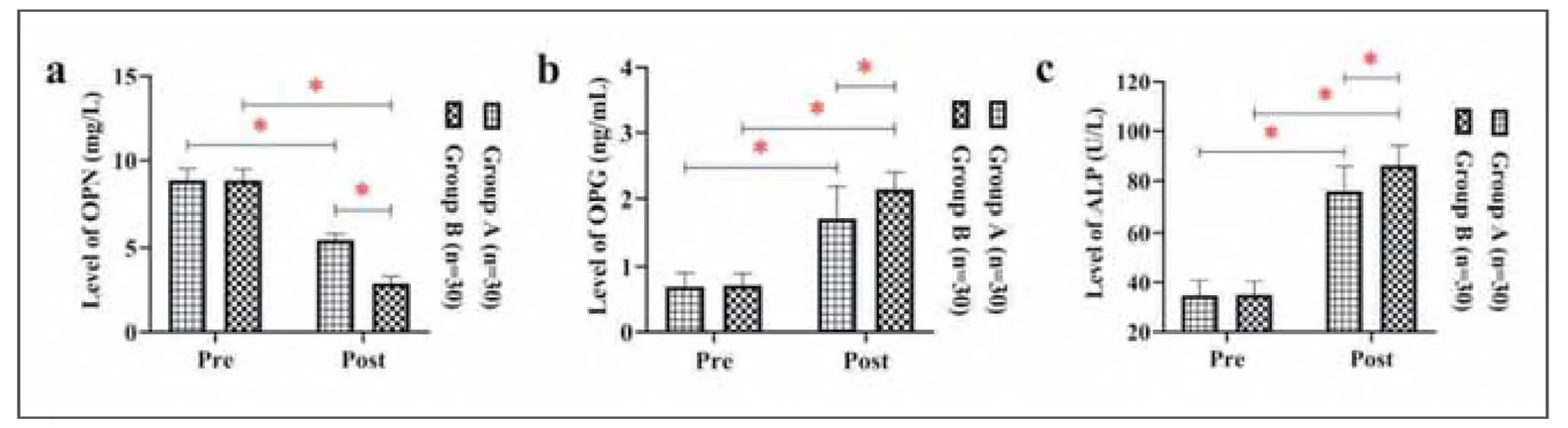
Comparison of bone metabolism indicators between the two groups. *P < 0.05. (a) Changes of OPN before and after treatment; (b) Changes of OPG before and after treatment; (c) Changes of ALP before and after treatment.

In terms of bone metabolism, both groups exhibited increases in post-treatment OPG and ALP and a drop in OPN (P<0.05). Further inter-group comparison indicated higher OPG and ALP levels as well as lower OPN concentrations in group B compared to group A (P<0.05), demonstrating better bone metabolism in group B (Figure 2).

### Comparative analysis of cell differentiation capacity

**Figure 3:**
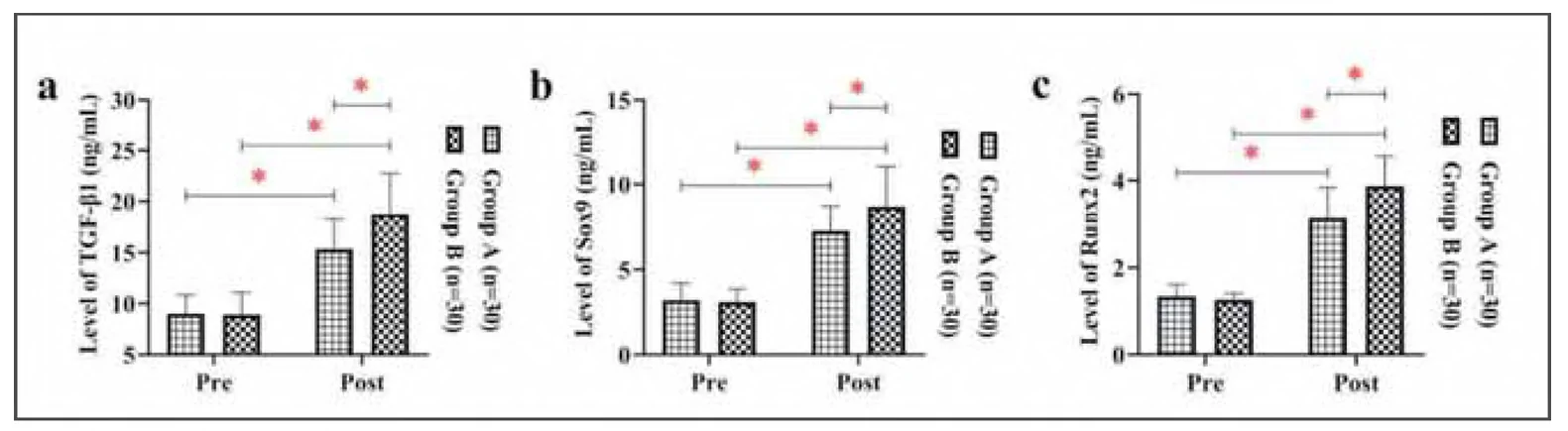
Comparison of cell differentiation indicators between the two groups. *P < 0.05. (a) Changes of TGF-β1 before and after treatment; (b) Changes of Sox9 before and after treatment; (c) Changes of Runx2 before and after treatment.

This study further analyzed changes in cell differentiation function pre- and post-treatment. In group A, Runx2, TGF-β1 and Sox9 increased after treatment (P<0.05). Elevations in Runx2, TGF-β1, and Sox9 were noted in group B compared to pretreatment measurements, higher than those in group A (P<0.05). The findings suggest superior cellular differentiation capacity in group B patients compared to group A (Figure 3).

### Comparison of coagulation function

**Figure 4:**
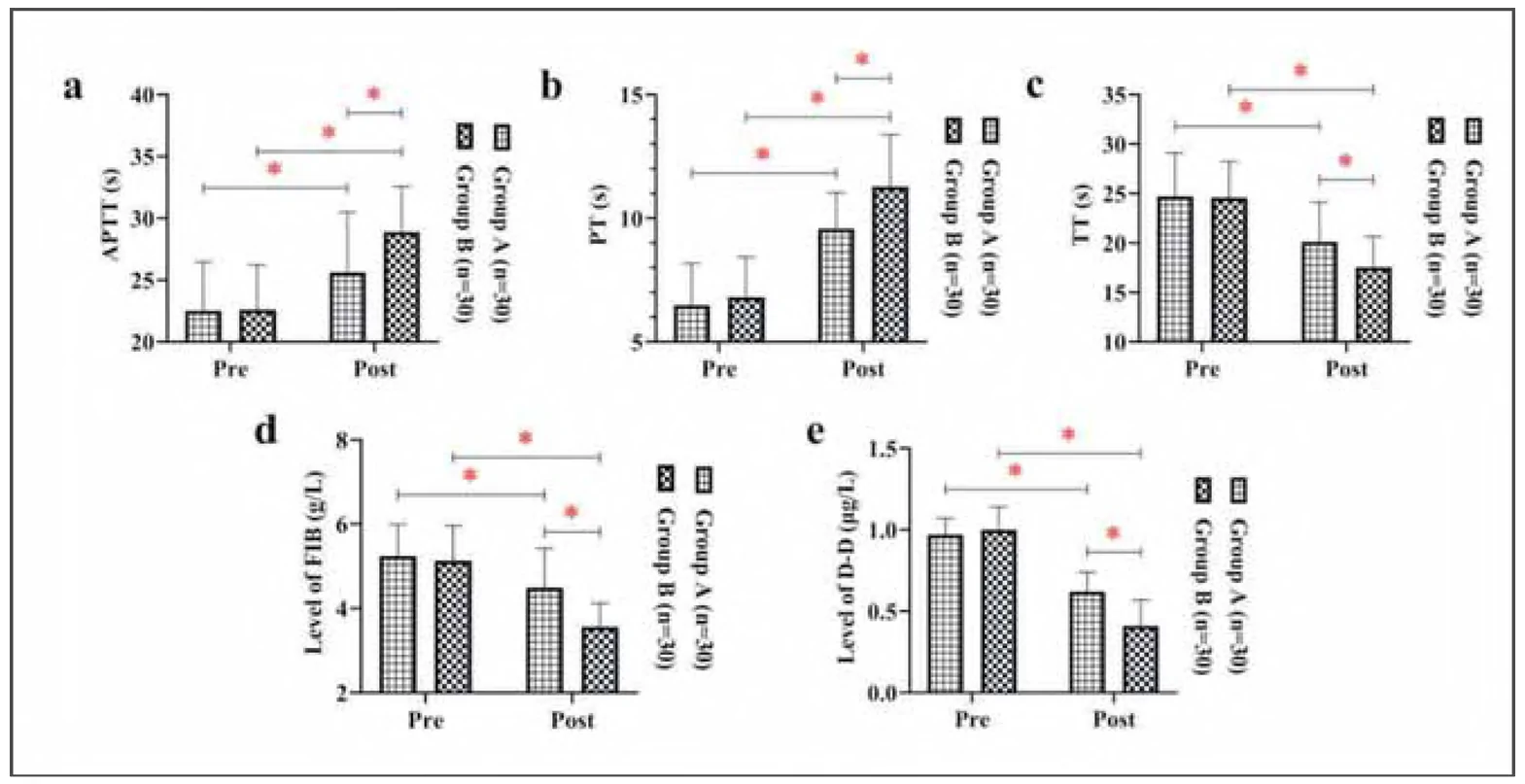
Comparison of coagulation function between the two groups. *P < 0.05. (a) Changes of APTT before and after treatment; (b) Changes of PT before and after treatment; (c) Changes of TT before and after treatment; (d) Changes of FIB before and after treatment; (e) Changes of D-D before and after treatment.

In coagulation function analysis, group A showed no significant changes post-treatment (P>0.05). In contrast, group B exhibited prolonged APTT and PT (P<0.05 *vs.* group A), while TT, FIB, and D-D levels decreased significantly (P<0.05 *vs.* group A). These findings confirm the alleviation of hypercoagulability in group B following treatment (Figure 4).

## Discussion

By comparing the effects of meloxicam + HCQ (group A) and its combination with HCQ (group B) in treating TMJ-OA, this study found that both treatments led to markedly reduced inflammatory factors (IL-1β, IL-6, TNF-α), ameliorated bone metabolism indexes (increased OPG and ALP and decreased OPN), enhanced cell differentiation (elevated TGF-β1, Sox9, and Runx2), and reversed hypercoagulable state (prolonged APTT and PT plus reduced TT, FIB, and D-D). Notably, group B showed superior performance to group A in inflammatory control, bone metabolism balance maintenance, and cell regeneration, suggesting that the combination of PRF with HCQ may offer greater therapeutic potential for TMJ-OA management through potential synergistic or additive mechanisms.

Chronic low-grade inflammation is one of the hallmark pathological features of TMJ-OA. Pro-inflammatory factors, represented by IL-1β, IL-6 and TNF-α, induce the secretion of matrix metalloproteinases (MMPs) via NF- B pathway activation, thus degrading cartilage matrix and inhibiting osteoblast differentiation [11]. In this study, both treatments induced marked reductions in inflammatory factor levels (P<0.05); However, the decrease was even greater in group B, possibly attributed to the synergistic anti-inflammatory mechanism of PRF and HCQ. PRF, as an autologous biomaterial abundant in endogenous anti-inflammatory factors like TGF-β1 and IL-10, can directly inhibit the abnormal activation of synovial fibroblasts and macrophages through paracrine action [12]. Being a classical immunomodulator, HCQ can block TLR signaling to reduce NF- B nuclear translocation, thus inhibiting proinflammatory factor transcription [13]. When used in combination, PRF's growth factor network and HCQ's signaling inhibition demonstrate complementary actions, enhancing anti-inflammatory efficacy through a multi-target approach consistent with Kargarpour Z et al.'s [8] theoretical framework.

In TMJ-OA, increased osteoclast activity (elevated RANKL/OPG ratio) leads to excessive bone resorption, while impaired osteoblast differentiation (reduced ALP activity) results in insufficient bone formation, ultimately causing bone spurs or cystic changes [14]. This study observed an elevation in OPG (a competitive inhibitor of RANKL) and a reduction in OPN (a protein involved in bone remodeling) post-treatment in both groups. Notably, group B exhibited more pronounced changes, indicating a shift in bone metabolism toward absorption inhibition and formation promotion. Bone morphogenetic proteins (BMPs) released by PRF can directly activate the osteogenic differentiation process of MSCs, up-regulate Runx2 expression, and promote ALP secretion [15]. HCQ reduces the ability of osteoclast precursor cells to differentiate into mature osteoclasts by inhibiting RANKL receptor expression [16]. In addition, PRF's fibrin network offers mechanical support to osteoblasts, whereas HCQ mitigates inflammation-induced cytotoxicity, collectively fostering a dynamic balance in bone repair.

Tissue regeneration impairment in TMJ-OA is characterized by suppressed chondrocyte differentiation (manifested as Sox9 downregulation) and hindered osteochondral differentiation potential of MSCs [17]. In the present study, while monotherapy (group A) elevated TGF-β1/Sox9, combination treatment (group B) additionally enhanced Runx2 expression. These findings indicate that PRF + HCQ combination therapy uniquely promotes both chondrogenic and osteogenic pathways. TGF-β1 serves as a master regulator of chondrocyte extracellular matrix synthesis, and its high concentration in PRF directly activates SOX9 transcription to restore chondrocyte phenotype [18]. Furthermore, PRF-released platelet-derived growth factor (PDGF) promotes MSC aggregation to injury sites [19]. Such coordinated regulation of chondro-osteogenic differentiation provides a novel cellular strategy for TMJ-OA regeneration.

In the local microenvironment of TMJ-OA, inflammatory factors like TNF-α can induce vascular endothelial cells to express tissue factor (TF), thus activating exogenous coagulation pathways and leading to local hypercoagulability [20]. When evaluating coagulation parameters, group B was found to show extended APTT and PT (reflecting decreased coagulation activity) coupled with diminished TT, FIB, and D-D levels (indicating fibrinolysis normalization), supporting the hypothesis that PRF-mediated inflammation suppression may beneficially influence coagulation mechanisms. Unlike conventional biomaterials, PRF's fibrin matrix demonstrates unique dual functionality: as a molecular sieve capturing inflammatory factors in the circulation and reducing their dissemination to the joint cavity and as a fibrinolysis promoter by providing binding domains for fibrinolytic enzymes [21]. The resulting coagulation-fibrinolysis rebalancing prevents local microthrombus-induced ischemic injury while creating a permissive environment for tissue repair via enhanced cellular infiltration and matrix accumulation.

Although our study initially confirmed the effectiveness of PRF + HCQ therapy, several limitations merit consideration: (1) The limited sample size, comprising only mild-to-moderate TMJ-OA cases, precludes generalization to severe patients; (2) The 3-month follow-up period is insufficient for evaluating long-term outcomes or recurrence risk. Future studies should incorporate longer follow-up periods (12 months) to evaluate the durability of the treatment effects and the risk of symptom recurrence; (3) Reliance on hematological parameters without assessment of synovial inflammatory markers (e.g., cytokines, MMPs) limits understanding of the articular microenvironment dynamics; (4) The study design did not include a PRF monotherapy group or an HCQ monotherapy group. Therefore, the observed superior effects in group B could be attributed solely to PRF, solely to the combined effect of the two drugs in group A, or to a true synergistic effect between PRF and HCQ. The specific contribution and potential synergy of PRF cannot be definitively established without additional control groups.

## Conclusion

This study confirms that PRF plus HCQ therapy can significantly alleviate inflammation in TMJ-OA, optimize bone metabolism, enhance cell differentiation, and improve coagulation function parameters through multi-pathway effects, potentially involving synergy or additivity between the components, including anti-inflammatory, tissue-reparative, metabolic regulatory, and anticoagulant properties, demonstrating far superior efficacy to PRF monotherapy. The findings provide a new strategic choice for the comprehensive treatment of TMJ-OA. However, further research with larger cohorts, extended follow-up periods, and multi-omics analysis is needed to elucidate the underlying molecular mechanisms and facilitate clinical translation.

## Dodatak

### Availability of data and materials

The data that support the findings of this study are available from the corresponding author upon reasonable request.

### Funding

Not applicable.

### Acknowledgements

Not applicable.

### Conflict of interest statement

All the authors declare that they have no conflict of interest in this work.
